# Bruxism and Botulinum Injection: Challenges and Insights

**DOI:** 10.3390/jcm12144586

**Published:** 2023-07-10

**Authors:** Giuseppina Malcangi, Assunta Patano, Carmela Pezzolla, Lilla Riccaldo, Antonio Mancini, Chiara Di Pede, Alessio Danilo Inchingolo, Francesco Inchingolo, Ioana Roxana Bordea, Gianna Dipalma, Angelo Michele Inchingolo

**Affiliations:** 1 Department of Interdisciplinary Medicine, University of Bari “Aldo Moro”, 70121 Bari, Italy; giuseppinamalcangi@libero.it (G.M.); assuntapatano@gmail.com (A.P.); c.pezzolla3@studenti.uniba.it (C.P.); l.riccaldo@studenti.uniba.it (L.R.); dr.antonio.mancini@gmail.com (A.M.); c.dipede1@studenti.uniba.it (C.D.P.); ad.inchingolo@libero.it (A.D.I.); angeloinchingolo@gmail.com (A.M.I.); 2 Department of Oral Rehabilitation, Faculty of Dentistry, Iuliu Hațieganu University of Medicine and Pharmacy, 400012 Cluj-Napoca, Romania; roxana.bordea@ymail.com

**Keywords:** bruxism, botulinum toxin, injection, type A, review, dentistry, masseters, temporalis, medial pterygoids

## Abstract

Botulinum toxin (BTA) is a bacterial-derived extract that can inhibit muscle contraction, acting directly on the absorption of acetylcholine. Thanks to this property, botulinum has been used in aesthetic and general medicine for several years. Nowadays, the use of botulinum toxin is being deepened to address the problem of bruxism. In this scoping review, the results of the studies in the literature of the last 10 years were analyzed. Indeed, 12 reports (found on PubMed, Web of Science, and Scopus, entering the keywords “BRUXISM” and “BOTULINUM TOXIN”) were deemed eligible for inclusion in this review. In the studies reviewed, BTA was injected into different muscle groups: masseters, masseter and temporalis or masseter, temporalis, and medial pterygoid. Botulinum toxin injection is a viable therapeutic solution, especially in patients with poor compliance or without improvement in conventional treatment.

## 1. Introduction

Bruxism is a visible habit of clenching or grinding teeth. It is a parafunction, or an activity different from swallowing, chewing, language, breathing, and all actions carried out physiologically by the chewing apparatus [1].

From the scientific literature, it seems that about 20% of the population suffers from day bruxism and about 10% from night bruxism [2]. In general, those who suffer from bruxism during the day tend to tighten their teeth rather than grind them, while, during night bruxism, the tightening and grinding of teeth are overlapping [3].

It is not always possible to identify a single etiological factor of bruxism, but some risk factors are known, such as anxiety and stress, mood and/or sleep disorders, taking certain drugs, such as antidepressants and antipsychotics, the abuse of alcohol or drugs, as well as smoking (it seems in fact that, in smokers, there is bruxism five times more than in the non-smoking subjects [4,5]. To date, there is no specific treatment for this disorder, but it is certainly possible to control its symptoms and reduce the risk of complications [6]. When bruxism is highly related to strong psychological discomfort, it is good to treat this disorder with the help of a professional, who provides supportive psychological therapy, as well as trying to work spontaneously on reducing the patient’s stress [7]. In most cases, it is useful to protect teeth during the night with a specific bite, a device that can absorb the pressure exerted by the jaw and act as a physical barrier between the dental arches [8]. Unfortunately, more and more often, we realize that the night bite is not enough and then resort to botulinum toxin, in particular type A toxin, to put the masseter to rest. In the case of bruxism, clinical experience shows how the use of about 12/18 U.I. of toxin, inoculated in the abdomen of the masseter, can, together with the bite, significantly reduce morning headache and put the muscle to rest, thus reducing hypertrophy [9].

Botulinum toxin (from the Latin word “botulus”, meaning “sausage”) is a bacterial-derived extract that acts at synapses by inhibiting the uptake of acetylcholine by neurons, thus causing inhibition of muscle contraction [10,11]. The application of botulinum in oral and maxillofacial surgery began in 1982 when Jan Carruthers began using it to reduce muscle mass and smooth the skin [12,13]. Since that time, the targeted injection of the toxin, considered a noninvasive procedure, has been used by aesthetic medicine and in the cosmetic field to manage frown lines [14,15].

However, its properties have proven particularly useful for various possible muscle spastic disorders, thus presenting the possibility of a multitude of applications in the treatment of complex disorders of the maxillofacial district, such as temporal mandibular disorders, lip hypertonicity, trigeminal neuralgia, and, not least in importance, bruxism [16,17].

### 1.1. Mechanism of Action of the Toxin

Botulinum toxin is extracted from the bacterium clostridium [13,18,19]. The toxins can be serologically differentiated into eight types, named A, B, C1, C2, D, E, F, and G, respectively. Among the eight types present, only the first two neurotoxins, A and B, are commercially available, specifically three forms of botulinum toxin A (BTA) (Botox^®^, Xeonin^®^, Dyspord^®^) and a single formulation of toxin B (Myobloc^®^) [4,9,10].

**Figure 1:**
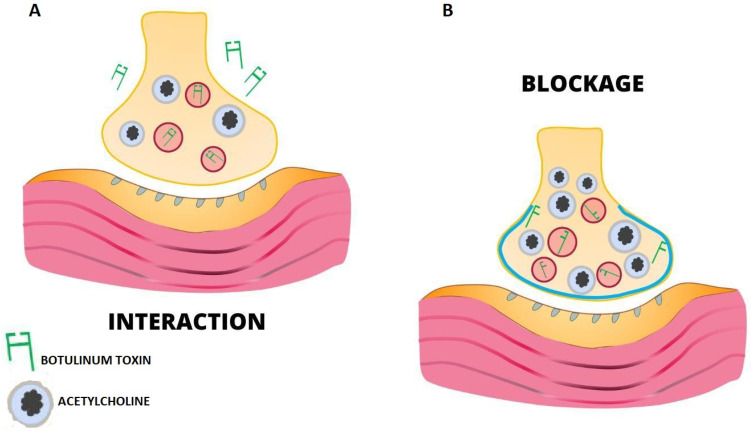
(**A**) The toxin binds via the heavy chain to presynaptic receptors. The light chain (the active chain) penetrates the cell through the disulfide bridges of the molecule. (**B**) ZN++-dependent peptidases; once the BTA molecule (light and heavy chains) is in the cytoplasm, break the disulfide bridges of the BTA. The light chains translocate inward to the cytosol and prevent the fusion of acetylcholine vesicles with the cytoplasmic membrane.

Botulinum toxin is approved by the DA for use in cases of cervical dystonia and hemifacial spasms [20,21,22]. However, although these two subtypes have antigenic variations between them, they have similar functions and can be used in an injectable form by intramuscular and intradermal means [23,24,25]. When the intramuscular route is chosen, the toxin causes proteolysis of SNAP-25, a synaptosomal-associated protein found in the neuronal cytoplasm that is essential for acetylcholine discharge at the neuromuscular junction. This resets the action potential to zero and muscle contraction is inhibited, resulting in paralysis of the affected muscle (Figure 1). The effect related to botulinum toxin gradually then degrades and disappears completely after 3 months [26].

### 1.2. Possible Fields of Application

The fields of application in the head and neck area where botulinum toxin can be used can be roughly divided into cosmetic and medical–therapeutic use.

#### 1.2.1. Cosmetic Use

**Figure 2:**
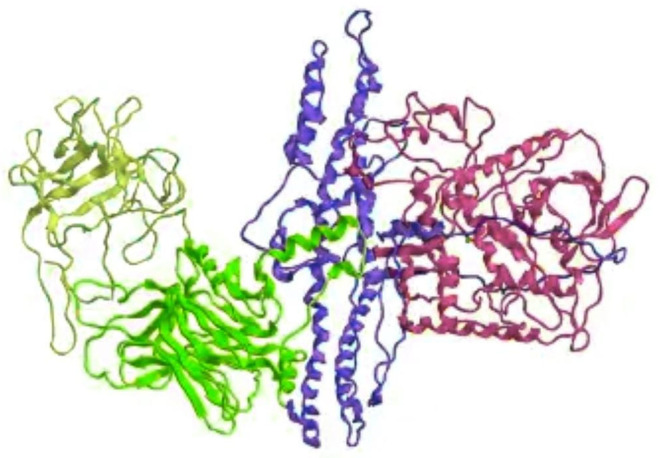
Botulinum toxin type A.

The botulinum toxin used in cosmetic medicine treatments is type A (Figure 2), and it is usually injected in the frontal–glabellar region and on the frontal–periocular surface, that is, the area surrounding the eyes. It is used to smooth out expression roughness, leonine folds interposed between the eyebrows, frontal subtalar roughness, crow’s feet, and gingival smile [27,28].

#### 1.2.2. Use of Botulinum Toxin against Wrinkles

Botulinum toxin reduces and modulates the muscular contraction of the mimic muscles, thus promoting skin relaxation [29,30]. Expression lines, depending on their depth, will improve until they disappear, and the action will be consolidated over time. Botox grants the possibility of obtaining a lifting effect of the tail of the eyebrow as well, thus providing the effect of raising the tail of the eyebrow and thus widening the gaze without resorting to blepharoplasty, although it does not replace it [31,32].

#### 1.2.3. Over-Exposure of the Gingival Portion

Botulinum toxin can be effectively used in individuals with gingival smiles in which there is excessive exposure of the maxillary gingival tissues during smiling. The exposure of the gingival mucosa plays a key role in the beauty of the smile [33,34]. When we have an overrepresentation of the rose portion, this may be due to a skeletal defect or lip muscle overactivity. In the second case, the conventional treatment turns out to be a surgical correction of the overactive labial muscle. This method, however, has a disadvantage, which is scar contraction and relapse in muscle activity [18,35]. However, there is a much less invasive approach that still offers an excellent method of correcting hypertonia: botulinum injection therapy [18,35].

#### 1.2.4. Medical–Therapeutic Use

**Figure 3:**
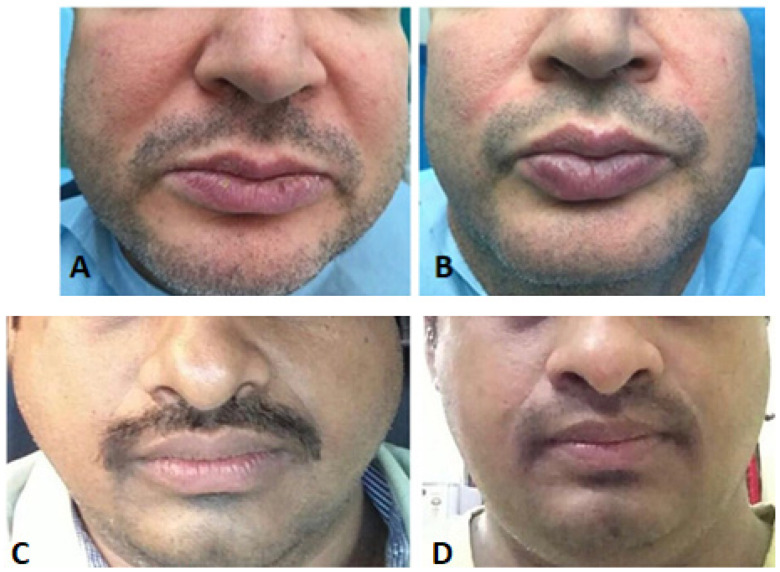
Two cases treated with masseter injections of Botox: (**A**,**C**), patient before the injection; (**B**,**D**), 40 days after the injection. It is evident how the masseters have normalized.

Therefore, in addition to its use in the field of aesthetic medicine, botulinum toxin can also be used as a drug in other fields of medicine to counteract disorders and pathologies in different districts of the body [36,37]. As mentioned, both botulinum toxin type A and botulinum toxin type B are used in the field of nonaesthetic medicine. Botulinum toxin B (Myobloc^®^) is approved by American Dentistry for use in cases of cervical dystonia and hemifacial spasms [38,39]. Type A, on the other hand, is indicated in:

- Treating persistent muscle spasms (post-apoplexy, blepharospasm, bladder hyperactivity…) [40];
- Chronic headaches (when botulinum toxin A injections were used in patients undergoing cosmetic treatment with an associated headache, improvement in chronic headache was observed [41,42];
- Hyperhidrosis (usually affects armpits, hands, and feet) [43,44];
- Trigeminal neuralgia. There are several case reports and studies demonstrating its effectiveness to reduce the symptoms of this disorder [45], such as numbness, tingling, and scratching sensation in the affected areas [46,47];
- Masseter hypertrophy (Figure 3). Usually, patients with a history of bruxism are affected, and the appearance of their faces may change due to the increased size of the muscle. In particular, the jaw appears swollen and malformed. The conventional treatment modality to follow is surgery, but, today, the minimally invasive technique is injection [48];
- Sialorrhea. Botulinum acts as an anticholinergic by reducing salivary secretions [24];
- Black triangles between prosthetic anterior teeth. Botulinum toxin is injected into the interdental spaces to replace lost interdental soft tissue, thereby improving the aesthetics of the prosthesis. The treatment effect lasts approximately 8 months [39];
- Temporomandibular disorders (TMD) and bruxism. TMD indicate a group of disorders related to joint and/or masticatory muscle dysfunction, such as joint clicks, headaches, joint and/or periauricular pain, neck stiffness, and associated pain. Occlusal disharmonies and periodontal problems play a major role in the etiology of TMDs. Some studies claim that botulinum toxin injection relieves associated joint pain and improves mouth opening [48,49].

Sleep bruxism (SB) is defined as a repetitive activity of the masticatory muscles characterized by clenching or wear of the teeth and/or increased mandibular muscle tone and is one of the most increasingly common pathologies in dentistry; it has an increasingly frequent occurrence as it is associated with daily parafunction, involving the masticatory joint, also generated by states of “stress” [50].

**Figure 4:**
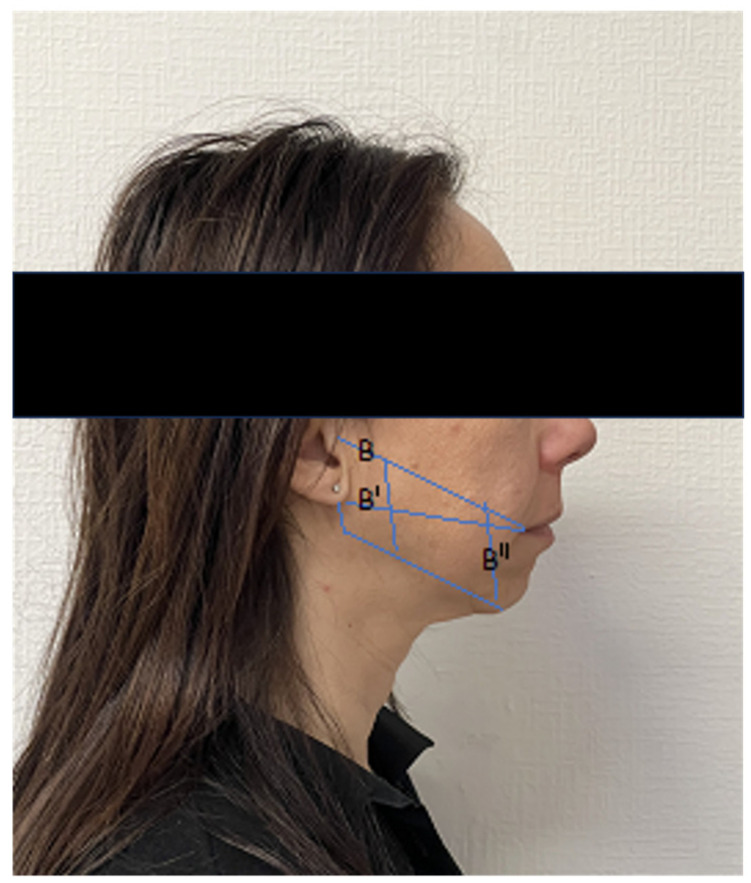
Markings for a single botulinum toxin injection point for masseter reduction in this 28-year-old female representative patient. Line B″ is drawn along the anterior border of the masseter with teeth clenched and the most prominent bulge of the muscle. Line B or B′, depending on individual anatomy, is then drawn towards the oral commissure to capture most of the masseter bulk.

Injection of BTA into the masseter (Figure 4) and temporalis muscles is proposed as a therapy for nocturnal bruxism as it exploits the effect of partial temporary muscle paralysis. Its effect lasts only a few months, and then everything returns to as before unless the therapy is repeated. A retrospective review has been published in the International Journal of Oral Maxillofacial Surgery concerning the clinical results obtained from botulinum toxin type A injections on the symptoms of temporomandibular disorders (TMDs) and bruxism. The authors conclude that BTN-A promises good results for the management of chronic diseases, such as TMDs, particularly in patients with masticatory muscle changes associated with psychiatric comorbidity [24].

The aim of this study is to evaluate the possibility of the use of botulinum in the treatment of bruxism, analyzing its advantages and disadvantages.

## 2. Materials and Methods

### 2.1. Protocol and Registration

The current review was carried out in compliance with the standards of PRISMA Extension for Scoping Reviews (PRISMA-ScR) [51].

### 2.2. Research Processing

We used PubMed, Scopus, and Web of Science as online databases, in which we searched for publications that matched the topic of the review. The search method was developed by combining phrases that matched the objective of our review, which is about the treatment of bruxism using botulinum toxin, examining the injection zone and the protocol of use. The keywords used for the analysis were “BRUXISM” and “BOTULINUM TOXIN,” and we used the word “AND” as a Boolean variable. The articles considered were published in English and referred to a time interval of 10 years, from January 2013 to January 2023 (Table 1).

### 2.3. Inclusion Criteria

The inclusion criteria were: (1) studies on humans, (2) open access, (3) English language (English abstracts and foreign language texts were found), and (4) comparison with other therapies.

### 2.4. Exclusion Criteria

The exclusion criteria were: (1) case report, (2) animals, (3) in vitro, (4) other reviews, (5) off-topic, and (6) bruxism induced by other conditions (e.g., Alzheimer’s, otalgia, brain injury, cerebral palsy).

### 2.5. Data Processing

We excluded articles that did not fit the topic by reading the manuscript title and abstract. The full texts of the remaining articles were read to assess the relevance based on the inclusion criteria. Disagreements between authors on article selection were discussed and resolved.

## 3. Results

A total of 370 articles were found by entering the keywords “BRUXISM” and “BOTULINUM TOXIN” in three databases, including PubMed (97), Scopus (164), and Web of Science (109), resulting in 194 articles after removing duplicates (176).

In the next step, reading the titles and abstracts led us to exclude another one-hundred-forty-seven articles (two animal text, sixty-nine reviews, and seventy-six off-topic).

Among the 47 articles selected, 11 texts were not open access, so there were 36 articles eligible for the eligibility phase.

**Figure 5:**
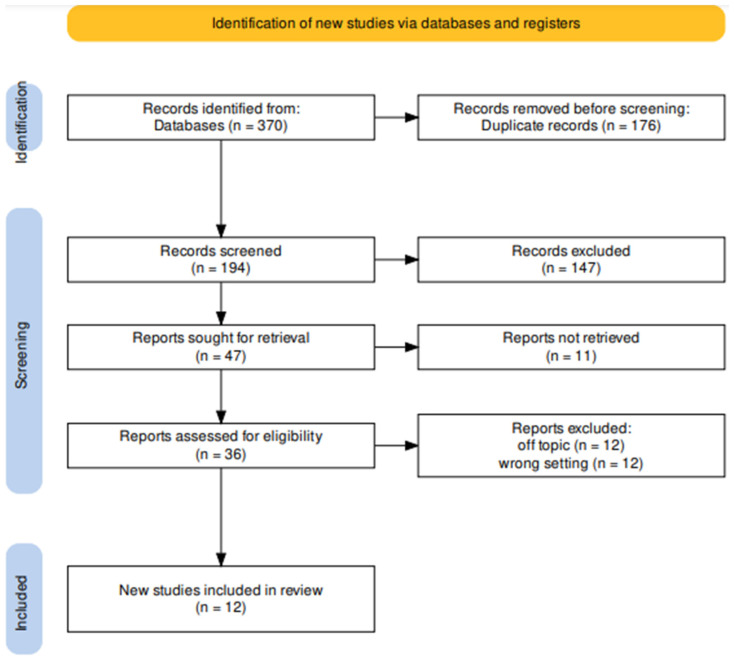
Literature search according to PRISMA Extension for Scoping Reviews (PRISMA-ScR) flow diagram.

The full texts were analyzed, and, once unrelated articles to the topic (12) and the wrong setting were excluded, 10 reports were deemed eligible for inclusion in this review (Figure 5).

## 4. Discussion

Bruxism consists of the repetition of afunctional chewing cycles, without the presence of the bolus, with muscle contraction. It may occur during night (sleep bruxism, SB) or day (awake bruxism, AB). A recent study stated that bruxism should not be considered a movement disorder but a “behavior” that predisposes to joint, dental, and prosthetic problems [52,53,54].

Clinically in patients, it is possible to observe tooth wear, tooth mobility, fracture or chipping of prosthetic restorations, indentations on the tongue, linea alba along the genial mucosa, masseterine hypertrophy, myalgia, and predisposition to TMD [55,56,57,58].

The pathogenetic mechanism of bruxism is still unclear; it is probably multifactorial and mediated by the central nervous system, with an important psychosomatic component [4,59,60].

Van Zandijcke and Marchau were the first to successfully use botulinum toxin for the treatment of bruxism in a patient with cerebral injury [61].

From this time on, the toxin has been used by other scholars to treat forms of bruxism secondary to other conditions or diseases, such as Huntington’s chorea, autism, orofacial dystonia, and the abuse of amphetamines [59].

The muscles involved in the jaw elevation movement are the masseter, temporalis, and medial pterygoid, which is why these three muscles are considered the sites of toxin injection in patients with bruxism (Table 2) [62].

### 4.1. Masseters

A comparison of outcomes between Saudi female patients treated with 20 U of BTA injected into three points of the masseter (group A) and patients treated with traditional methods (i.e., cognitive–behavioral therapy, drug therapy, occlusal splints) (group B) showed that, even after one year, significant differences persisted between group A and B after injection in terms of pain perception. The mean pain score (MPS) in group A after one year was 0.2 ± 0.51, and, in group B, it was 2.1 ± 0.74 (Table 3) [65].

In Shim’s study, patients who were already being treated with occlusal splints but were not finding benefits and had wear on the splint itself were analyzed. The post-injection polysomnographic evaluation confirmed that botulinum reduces the intensity of masseter muscle contraction for at least 12 weeks but does not change the frequency of occurrence of RMMA episodes [72]. On the other hand, the author offers a different perspective of bruxism: RMMA are physiological episodes, so there is no need to intervene numerically in these in trying to reduce them [73]. Instead, the clinician’s goal is to reduce the intensity of the contraction of the muscles involved in this phase: it is achieved by injection, as is possible [52].

Ultrasonography could be a useful tool to evaluate how long to repeat botulinum toxin injection [64]. In 43 female patients reporting algic symptoms, in the follow-up appointment after the injection at three sites in the masseter, it was observed that 26% of patients no longer experienced symptoms of bruxism, while the remaining 74% noticed a decrease in pain or muscle tension according to a scale of 0 to 4 of personal satisfaction with the treatment (0 = no improvement; 4 = no symptoms) [64].

Ultrasound evaluations of outcomes four months after the first injection and five months after the second one are important since, when the values become closer to the base value, a next appointment should be scheduled after 4 weeks, thus preventing loss of efficacy. Similarly, an assessment of muscle thickness using a caliper showed that muscle thickness is directly proportional to the patient’s reported symptoms: after injection, masseter thickness decreases and, likewise, symptoms disappear or decrease. Facial aesthetics also improves due to reduced thickness and muscle relaxation [64].

Moreover, after approximately 5 months (4.76 ± 1.01), a reduction in the effect of 20 U of BTA injected at four sites in the masseter was observed. In this timeframe, in all patients, an improvement in symptomatology assessed by VAS was shown, while two patients did not notice any improvement at all: the VAS score remained the same as the pre-treatment value [63].

Changes in voluntary mouth opening also did not show statistically significant differences, unlike when studied by Sidebottom et al. and Guarda-Nardini et al. [59,74,75].

With a lower dose of BTA (10 MU of BTA per side at two sites in both masseters), the onset of loss in drug efficacy begins to occur earlier, after 3.51 ± 0.36 months. Despite the lower dose, the results demonstrated, as in those of other studies, changes in VAS values that proved consistent with other studies, with a peak pain reduction after 2 weeks and a subsequent progressive return to baseline from 4 months (probably due to the lower dose administered) [65,76]. Moreover, at EMG, the values between the control and placebo groups returned to similar 6 months after treatment; by 12 weeks, the electromyographic activity of the control group was significantly reduced [63].

### 4.2. Masseters and Temporalis

When a single muscle injection is a clinical strategy, there is a clinical indication that bilateral BTA injections into the temporalis muscles should be the first line of advice for treating SB [67,77].

One patient was evaluated by administering half the dose recommended in the literature: masseters received 15 U and the temporal 10 U, for a total of 50 U of BTA. A customized occlusal device instrumented at the level of the upper first molars with FBG was fabricated. FBG are optical fibers that allow evaluation of temperature, pressure, and strain using sensors [78]. As parametric changes occur, there is a change in the refractive index of the sensor in the fiber core, which is transmitted to a computer, and the value is interpreted by means of a color change. Using this method, the change in clenching force and sleep hyperactivity after toxin injection were analyzed [79]. Checks were carried out 45 days before cough application and after the 15th, 21st, one month, about two months, 105, and 134 days after injection. The patient had to hold the clenching position for 5 s. and then relax the muscles for 30 s. before starting again with a new cycle [69]. At the one-month follow-up appointment, there was the maximum peak reduction in masticatory strength; as early as the second month, after a single injection, there was an onset of recovery to normal activity (about 76% of the pre-injection baseline), reaching 90% of the initial value after 130 days. Sleep hyperactivity tests were completed with the patient sleeping in the laboratory in three steps: before the injection and 4 and 8 weeks after the injection of BTA. Before the injection, in a period of one hour, four spikes of hyperactivity were recorded, interspersed with about 10 min of relaxation between each spike; after one month following the injection, the time interval lengthened to 20 min, effectively reducing the number of spikes per hour (three spikes). After two months, however, there was a return to a value of four spikes, and the intervals between spikes averaged 15 min [80].

After a polysomnographic evaluation, twenty-three subjects were taken into analysis: thirteen subjects received botulinum toxin injection (100 U/mL) at three sites in the temporal (40 units) and two in the masseters (60 units) for a total of 200 units; ten subjects received placebo at the same sites. Clinical global impression (CGI) and VAS for bruxism and pain were assessed before injection and between 4 and 8 weeks after injection by repeating PSG the night before the appointment [70].

Although polysomnographic evaluation showed no significant differences between the two groups, there was an improvement in sleep duration in the control group (increased 34.3 ± 58.6 min vs. decreased 11.7 ± 53 min in the placebo group). The number and duration of bruxism episodes were also reduced in the control group. In addition to these values, additional scales, such as exploratory scales, including the Headache Impact Test-6, total Pittsburgh Sleep Quality Index, Epworth Sleepiness Scale, and Self-Rated Anxiety Scale, were not significantly changed [70].

In contrast, VPSG evaluation and simultaneous EMG recordings of masticatory muscles following injection of BTA revealed a reduction in the intensity of muscle contraction during sleep, although it did not change the frequency of RMMA episodes. Patients in the study received a bilateral injection into the masseters or the masseters and temporalis simultaneously. Each muscle was stung at three sites and 25 U of the toxin was injected. Patients were analyzed the evening before injection and 4 weeks after treatment. Nine patients, with a higher RMMA baseline index than the other subjects, reported a reduction in tooth grinding; the other patients did not report any changes. Morning stiffness was also reduced after injection in both groups (47.50 ± 15.86% in group A and 57.50 ± 30.30% in group B). 

Electromyographic evaluations, on the other hand, showed a clear reduction in maximum masticatory force and duration of RMMA episodes in the injected muscles, confirming that it is the force of contraction that is reduced and not the number of episodes. Nevertheless, except for a spike in the EMG burst of the temporalis muscle in group B after injection, there were no statistically significant differences between the two groups. It is plausible to think that the two muscles contract synergistically during the closing phase, so the injection of BTA has a cumulative effect related to the higher dose introduced into the body, reducing the total force generated [67].

Da Silva Ramalho et al. recruited 20 subjects reporting facial pain correlated to the bruxism condition for at least 6 months. In a randomized single-blind clinical trial, they compared the injection of BTA into the masseters (group A) and into both masseters and temporalis muscles (group B) to analyze whether it was sufficient to inject only the masseter or was necessary to involve the temporalis muscle as well. The masseters were pinched in both groups at three points, and temporalis in group B was pinched at two points bilaterally. Injection with 10 U occurred for each point. Evaluations were carried out after 15, 90, 120, and 180 days; regarding pain perception, as early as 15 days after injection, patients reported a subjective improvement in painful symptoms (assessed by VAS), although not statistically significant; a reduction in bite force was also noted, assessed by placing a force transducer connected with a gnathodynamometer at the level of the molars. In general, patients were satisfied with the treatment, although there was no statistical difference between the two groups [66].

Yurttutan et al. demonstrated that botulinum toxin can be an effective solution in cases of patients poorly compliant with classical conservative therapies with occlusal splints [71]. Patients recruited in the study reported chronic pain for at least 6 months and were diagnosed according to Axis I of the RDC/TMD diagnostic criteria; 32 patients (group 1) received conservative treatment with occlusal splinting, 31 patients (group 2) received botulinum toxin injection bilaterally at five sites in the masseter muscle (30 U) and three in the temporalis muscle (15 U) for a total of 90 U, and 31 patients (group 3) received both treatments combined. Patients were administered questionnaires (TMD pain screener, Graded Chronic Pain Scale, Oral Behavior Checklist (OBC), and Jaw Function Limitation Scale (JFLS)) and performed muscle palpation to assess pain (using the VAS scale from 0 to 10) before treatment and six months later. The final evaluations showed that, in all three groups, there was an improvement in algic symptoms, but botulinum toxin injection provided better results overall than single occlusal splint treatment. Furthermore, in patients who underwent the combined treatment, there were no statistically significant improvements compared with botulinum toxin injection alone [71].

In Hosgor’s study, forty-four patients were divided into two groups and received botulinum toxin injection at three sites in the masseter and two in the temporalis (group A) and saline injection as a control at the same sites (group B) [81]. Then, VAS and a full range of motion were evaluated prior to treatment and in follow-up visits after 1 month, 3 months, and 6 months. The level of pain and VAS decreased significantly at follow-up visits, while the range of lateral and protrusion movements expanded due to less muscle distress. The psychological component of self-correction could also influence the reduction in parafunction [68].

### 4.3. Masseters, Temporalis, and Medial Pterygoids

In the study by Cruse et al., the medial pterygoid muscle was also considered as the injection site, in addition to the masseter and temporalis. Patients were divided into three groups: group A (six participants) received an injection in the masseter muscle (30 U), group B (seven participants) received the injection in the masseter (30 U) and temporalis (15 U), and group C (nine participants) instead in the masseter (U), temporalis (15 U), and medial pterygoid (15 U). Assessments were completed both objectively by electromyography for three nights and subjectively by administering questionnaires on reported symptomatology after 4 and 12 weeks. The group in which the injection was also administered in the medial pterygoid after 4 weeks showed a greater reduction in the Bruxism Index (BI) than the other two groups. In contrast, after 12 weeks, there were no significant differences in any of the three groups, regardless of dose or muscles injected, probably due to recovery of motor function after 3–4 months following injection. Furthermore, patients in all groups starting from a higher baseline BI achieved the best results after 4 weeks [9,82].

### 4.4. Collateral Effects

Side effects were found in patients in all studies reviewed, in contrast to the results of the studies by Shim et al. and Al-Wayli [63,65,67].

The most frequent cause of discomfort was perceived pain at the injection site, especially in patients in whom multiple muscle groups were treated, and in some cases lasted up to a week post injection with accompanying ecchymosis [59,63,64].

Only one patient decided to drop out of the study because of too much pain perceived after the first injection [9]. In some studies, xerostomia, speech problems, muscle weakness at the injection site, weakness and difficulty in swallowing (dysphagia), and bruising were reported as side effects, while, in other patients, there was only mild asymmetry of the lower third of the face and changes in the smile [66,70,71]. In the study by Asutay, the patients did not develop xerostomia as a symptom [59]. However, four participants developed masticatory weakness, and chewing pain resolved in two weeks; after the study ended, they still requested to continue with open label injections because the benefits were greater than the temporary complications [64]. Of fourteen patients who reported muscle weakness, only three actually developed real chewing difficulty (well tolerated), which resolved between 4 and 12 weeks [67]. Although it could occur as a complication of an allergic reaction, in no study was it evidenced [68,83,84,85]. In all patients, the complications resolved independently within a relatively short time frame (1 to 4 weeks), leaving almost no patients dissatisfied with the treatment [9,66,70,75].

Limitations observed during studies’ analysis are related to off-label use of BTA, since there are no standardized doses or preparation; the number and location of injection sites differ in the studies; the patients in the samples vary in age group, gender, and reported symptoms; the method of evaluation differs, in some cases relying only on questionnaires and subjective assessments; in others, these were supported by objective data recorded by EGM and PSG.

## 5. Conclusions

The masseter, temporalis, and medial pterygoid muscles are the jaw-elevating muscles, all involved in the para-physiological phenomenon of bruxism.

After injection, both subjective evaluation (questionnaires and VAS) and objective evaluation (PSG and EGM) show improvement in symptoms and muscle contraction status. Involvement of more sites or more muscle groups (and thus a higher final dose) lead to better and longer-lasting results, at least for the first month of action.

Further studies are needed to evaluate the phenomenon of addiction, necessitating higher dosages over shorter periods. In general, it was observed that injection should be repeated at least after about 6 months after the previous application.

In conclusion, botulinum toxin injection emerges as a viable therapeutic solution, especially in the case of patients who are poorly compliant or who have not noticed an improvement in symptoms following other treatments with conventional methods, despite the high cost and temporary discomfort.

Future studies should consider and unify techniques and dosages to standardize the procedure as its use in dentistry is now no longer “off label”.

## Figures and Tables

**Table 1:** Database search indicators.

Article searching strategy	Database: PubMed, Scopus, Web of Science
	Keywords: A “BRUXISM” and B “BOTULINUM TOXIN”
	Boolean variable: AND
	Timespan: 2013–2023
	Language: English

**Table 2:** Studies’ characteristics.

AUTHOR	TYPE OF STUDY	AIM	METHODS	RESULTS
Shim et al. [52]	Randomized, pacebo-controlled Trial	The aim of the study is to analyze the effects of BTA for managing SB	Thirty SB subjects were randomly assigned into two groups evenly. The placebo group received saline injections into each masseter muscle, and the treatment group received BoNT-A injections into each masseter muscle. Audio–video–polysomnographic recordings in the sleep laboratory were made before, at 4 weeks after, and at 12 weeks after the injection	SB episodes cannot be reduced with a single injection of BTA. Yet, by lowering the masseter muscle’s activity, it may be a useful management strategy for SB
Shehri et al. [63]	Randomized controlled trial	The aim is to assess whether BTA injections into the masseters reduce SB	Twenty-two subjects reporting SB were randomly divided into two groups: the control group received 10 MU of BTA in the masseters and the placebo group a sham intervention. To determine if this treatment approach was beneficial for SB, pain perception was measured using visual analogue scales (VAS) and muscle activity using electromyography (EMG)	20 patients completed the study. There were statistically significant variations in the control and placebo groups’ VAS before and after the injection (*p* ≤ 0.05). BTA injection reduces the strength of masseters contraction, thus providing relief from algic symptoms
Mkhitaryan et al. [64]	Prospective longitudinal study	The aim is to evaluate symptoms’ variation after BTA injection	Forty-three female patients were recruited in this study. Assessment controls were carried out before, two weeks, four months, and five months after the first BTA inoculation, as well as two weeks and five months after the second injection. The study was supported by photographs, orthopantomography, measurements of masseteres by means of a calliper, and ultrasound	After BTA injection, 26% of patients were free of symptoms and 74% declared a reduction in pain and correlated symptoms. Side effects were transitory and well tolerated. BTA injected in masseters was able to reduce discomfort and other symptoms while also preventing lesions on orofacial tissues
Asutay et al. [59]	Retrospective study	The objective of the study is to evaluate the effectiveness of BTA in the treatment of SB	25 female patients received BTA injection in both masseters and were compared regarding VAS values and effectiveness beginning and duration before and after the treatment	The pain scores significantly improved after using BTA. Only 2 negative impacts were noted. The medication BTA is efficient for SB
Al-Wayli et al. [65]	Randommixed clinical trial	The goal of the study is to compare BTA to other conventional methods for the treatment of symptoms correlated to SB	Patients were checked after three weeks, eight weeks, six months, and one year after the injection of 20 U, and the results were utilized to quantify the number of bruxism occurrences. A questionnaire was used to assess the symptoms	Botulinum injection reduces the intensity of contraction of muscle, thus reducing the pain score more than conventional treatments
da Silva Ramalho et al. [66]	Randomized clinical trial	The purpose of the study is to assess clenching force, face’s pain, and general relief of symptoms using two different protocols of BTA injection	BTA was injected randomly in 2 groups of patients: group A received the injection into the masseters (3 points in each muscle, 10 U per point) and group B was injected still in the same 3 points in masseters and 2 points in each temporal muscle (10 U per point). The patients were monitored before the injection and after 15, 90, 120, and 180 days with VAS, general satisfaction, and a dynamometer for muscle strength	10 patients for each group completed the study. In comparison to the baseline, both groups showed pain reduction after 15, 90, 120, and 180 days. Posterior bite force shows a decrease only till day 120th. Both groups were very satisfied in every follow-up appointment. In all evaluations and study periods, there were no differences between the groups
Shim et al. [67]	Randomized clinical trial	The aim is to study the effects of BTA injection in bruxer patients with or without orofacial pain not responding to oral splint therapy	This study was completed by twenty participants. BTA (25 U per muscle) was bilaterally injected into 10 subjects in only the masseter muscles (group A), while the remaining 10 individuals were injected into masseter and temporalis muscles (group B) with the same dosage. Videopolysomnographic (vPSG) recordings were taken prior to the injection and four weeks thereafter. The masticatory muscles’ regular movement orofacial activity (OFA) and rhythmic masticatory muscle activity (RMMA) were recorded and examined for various parameters (e.g., number of episodes, peak…). The electromyographic activity of the two muscles was also recorded	The two groups did not experience any differences in the frequency, quantity, or length of RMMA episodes in response to BTA injection. In both groups, the injection reduced the peak amplitude of EMG burst of RMMA episodes in the injected muscles. 9 individuals reported less teeth grinding one month after injection, while 18 subjects reported less morning jaw stiffness. Conclusions: SB can be effectively controlled with just one BTA injection for at least a month. Instead of reducing the frequency of activation, the botulinum reduces the intensity of contraction
Hosgor et al. [68]	Randomized clinical trial	This study evaluates the efficacy of BTA injection into the masseter and temporal muscles in patients with algic symptoms and SB	44 patients were evaluated before and after toxin injection in the masseters and temporalis muscles (after one, three, and six months) by administering VAS questionnaires and clinical measurements (e.g., maximum mouth opening, range of voluntary non-painful movement…)	Compared with baseline, patients’ perceived pain was significantly reduced and their range of motion expanded, making BTA a valid solution for SB and pain
Fontenele et al. [69]	Case study	The purpose of the study is to evaluate the intensity of clenching after BTA injection by means of a device instrumented with optical fibers	The patient was monitored during sleep while wearing an interocclusal device instrumented with fiber Bragg grating. Data transmitted to the software via sensors before and after toxin injection are compared	The confrontation of the value demonstrates a reduction of 25% in muscle hyperactivity and a lengthening of the parafunctional activity-free interval. Compared with other devices, the values obtained for data processing are more reliable, so a device equipped with optical fibers is a good tool for the clinician to evaluate this kind of improvement
Ondo et al. [70]	A double-blind, placebo-controlled study	The study wants to assess the treatment with BTA in patients with SB	Bruxers patients received injections in masseters (60 U for each) and temporalis (40 U for each) with BTA (control group) or saline solution (placebo group). Patients took questionnaires and tests to assess variation between before the inoculation and after 4 and 8 weeks from the injection; polysomnography and EGM were also recorded	Despite two patients reporting temporary smile changes, there were no significant differences in the analyzed parameters, the patients refer to improved sleeping duration and reduction in bruxism episodes, making BTA a valid therapeutic solution
Sancak et al. [71]	Pilot study	The study compares the outcomes after the application of occlusal splint and BTA in patients with bruxism	Seventy-three patients were randomly divided into 3 groups. Group A was treated with an occlusal device, group B was treated with BTA injection, and group C was treated with both options simultaneously. Before and six months following therapy, all individuals were administered the Temporomandibular Disorder Pain Screener, Graded Chronic Pain Scale, Oral Behavior Checklist, Jaw Function Limitation Scale, and VAS	The questionnaire and VAS scores decreased in all 3 groups. In patients treated with botulinum toxin (singly or together with occlusal splint), a better response was evidenced than treatment with splinting Thus, patients treated with BTA do not need adjuvant treatment
Cruse et al. [9]	Double-blind, randomised, placebo controlled crossover study	The goal of the study is to evaluate SB treatment with BTA	In the study, 41 subjects were divided and received injections of BTA in different muscles: group A 60 U in bilateral masseters, group B 90 U in masseters and temporalis, and group C 1200 U in masseters, temporalis, and medial pterygoid. The authors assessed changes in pain, bruxism, and headache at one month and three months after injection	SB can be treated safely and effectively with BTX-A. The administration of BTX-A into more muscles, at larger total dosages, and among those with higher baseline may result in a greater effect

**Table 3:** Mean pain perception in groups A and B.

	GROUP A	GROUP B
Pre-injection	7.1 ± 0.72	7.5 ± 0.66
3 weeks post injection	4.6 ± 0.58	5.4 ± 0.58
1 year post injection	0.2 ± 0.51	2.1 ± 0.74

## Data Availability

Not applicable.

## List of Abbreviations

BI	bruxism index
BTA	botulin toxin A
CGI	clinical global impression
EMG	electromyography
FBG	fiber Bragg gratings
JFLS	Jaw Function Limitation Scale
OBC	Oral Behavior Checklist
PSG	polysomnography
RMMA	Rhythmic masticatory muscle activity
SB	sleep bruxism
TMD	Temporomandibular disorders
VAS	visual analog scale
VPSG	Video polysomnographic
